# Exploring agrobiodiversity for nutrition: Household on-farm agrobiodiversity is associated with improved quality of diet of young children in Vihiga, Kenya

**DOI:** 10.1371/journal.pone.0219680

**Published:** 2019-08-02

**Authors:** Francis Odhiambo Oduor, Julia Boedecker, Gina Kennedy, Céline Termote

**Affiliations:** 1 Healthy Diets from Sustainable Food Systems Initiative, Bioversity International, Nairobi, Kenya; 2 Healthy Diets from Sustainable Food Systems Initiative, Bioversity International, Rome, Italy; Universidade de Sao Paulo, BRAZIL

## Abstract

Due to their limited access to the external productive inputs and the dependency on rain-fed agricultural production, small scale farmers in sub-Saharan Africa have continued to face undernutrition despite the significant advancements in agriculture. They however often live in areas endowed with high agrobiodiversity which could contribute, if explored, to improved diets and nutrition. Few studies have linked the contribution of agrobiodiversity to the micronutrient adequacy of the diets of young children among smallholder farmers. The study explored this relationship and contributes to the growing body of literature linking agrobiodiversity to nutrition of young children. Two cross-sectional surveys were conducted as part of baseline assessment for an intervention study, one in the lean and a second in the plenty season in Vihiga county, Kenya. Household level interviews were administered to 634 households with children 12–23 months. Agrobiodiversity was defined as the number of crop species cultivated or harvested from the wild and the number of livestock maintained by the household across two agricultural seasons. Dietary data were collected using two-non-consecutive quantitative 24-hour recalls and analyzed using Lucille software. Diet quality was assessed using dietary diversity score based on seven food groups and mean probability of micronutrient adequacy computed for eleven micronutrients. A total of 80 species were maintained or harvested from the wild by the households. Mean household species richness was 9.9 ± 4.3. One in every four children did not meet the minimum dietary diversity score. The average mean probability of micronutrient adequacy was 68.11 ± 16.08 in plenty season compared to 56.37± 19.5% in the lean season. Iron, zinc and calcium were most limiting micronutrients in the diet, with less than 30% average probability of adequacy in both seasons. Household agrobiodiversity was positively associated with both dietary diversity score (r = 0.09, p = 0.029) and micronutrient adequacy (r = 0.15, p<0.000) in the pooled sample. One unit increase in species diversity was associated with 12.7% improvement in micronutrient adequacy. Despite the rich agrobiodiversity in the study area the diets were low in diversity and there is an unrealized opportunity to improve micronutrient intake through greater promotion and consumption of locally available agrobiodiversity.

## Background

Malnutrition is among the most widespread causes of human suffering throughout the world [1]. Adequate nutrition in utero and in the first 2 years of life is essential for human capital development. Undernourished children are more likely to become short adults, have lower educational achievement, give birth to smaller infants, and have lower economic status in adulthood [2]. It is furthermore suggested that the effect of undernutrition spans at least three generations[2].

Despite the consequences, child undernutrition is still common in Kenya. According to the latest national survey, 26% of the children under-five years are stunted with children 18–23 months most affected at a prevalence rate of 35.5%. A further 4% of the under-fives are wasted and 5.9% are underweight. The report shows that 23.5% of children in Vihiga County are stunted [3]. A number of other smaller studies among Kenyan children have shown that children’s diets are limited in variety, diversity and nutrient composition and could be among the causes of the high stunting rates. For example, in western Kenya, one study based on a one week recall showed that only 3% of the children had consumed highly diversified diets while 45% of the preschool children had very low dietary diversity [4]. The findings indicated that 21.5% of the preschool children had not consumed any pulses or nuts, 11.8% had not consumed any meat or meat product, and 30.6% had not taken any milk or milk product during the one-week period. These results have been confirmed by yet another study in western Kenya which found that 40% of children in Vihiga did not meet the recommended minimum diet diversity [5]. Together, these studies confirm that diets of the Kenyan children are of poor variety, diversity and nutrient quality.

Agrobiodiversity, both wild and domesticated, can contribute to human nutrition in a number of ways including providing a rich source of nutrients for adequate dietary diversity and quality, improving farm resilience and income, providing a safety net against seasonal food shortages and hunger, and providing the genetic resources for future adaptation to eg. climate change [6–10]. Over the past decades, agricultural production based on a continuing and increasing dependence on external inputs has substantially increased food availability and access for the world’s increasing population[11]. But the production gains did not automatically translate into equally large nutritional gains. The highly input dependent agricultural systems tend to rely on a narrow diversity of crops and animals [9–10], often have detrimental effects on the environment and climate and are therefore unsustainable in the long run [11]. Moreover, more than 80% of the farmers are poor smallholders with limited access to these external inputs [11]. The net effect is the erosion of biodiversity, reduced variety of foods in the food baskets and high numbers of undernourished small holder farmers[12–13].

Despite the great potential of agrobiodiversity to improve diet quality, farm sustainability and resilience, its use remains underexplored especially among the rural smallholders [8,14–15]. Studies so far, have mainly focused on linking agrobiodiversity to household food security [16–17], household dietary diversity [14,16,18],woman’s or children’s dietary diversity [16, 19–20] and few to micronutrient adequacy [21]. This paper contributes to the available evidence linking agrobiodiversity to nutrition by exploring the association between on–farm biodiversity and the micronutrient quality of children’s diets among smallholder rural farmers in Vihiga County.

## Materials and methods

### Description of the study setting

The study was conducted in Vihiga county, western Kenya. With a total area of 531 square meters, the county is divided administratively into 3 sub-counties (Vihiga, Hamisi and Emuhaya), 9 divisions, 37 locations and 129 sub-locations [22]. The area belongs to two main agro-ecological zones, the upper and lower midlands. The county experiences two rainy seasons with average rainfall between 1800 and 2000 mm and the mean temperature is 23°C (range: 14°C–32°C). These agro-ecological conditions favor two planting seasons. The high rainfall supports high species diversity and endemism which results from the mixture of habitat types [23]

Agriculture, dominated by small scale farmers, constitutes 70% of the economic activities in Vihiga County with crop farming contributing to 64% of the county’s income. Maize and beans are the main subsistence crops while tea and coffee are the main cash crops. Other crops are sorghum, millet, cassava, sweet potatoes and bananas. The average farm size in Vihiga County is 0.4 ha for small farmers and 3ha for large scale farmers. The main livestock kept are cattle and chicken [22].

Vihiga County has a population size of 554,622 and a population density of 1045persons per square kilometer. Children 0–14 years of age constitute 45% of the population[24]. 62% of the population live below the poverty line [25] and the prevalence of stunting among under-five year old children is 23.5% [3].

### Sampling and study design

Data from surveys collected as part of the baseline assessment at the start of a project with aim to improve dietary diversity of women and children using locally available agrobiodiversity, were used for this study. Two cross-sectional surveys coinciding with plenty and lean seasons were respectively conducted in September–October 2014 and in March–April 2015 with no intervention between the two seasons. A minimum sample size of 400 per survey round was calculated using the FANTA published formula [26]. The indicator used was the proportion of children reaching minimum dietary diversity score (MDDS), adopting the 62.4% obtained from a previous study conducted by Bioversity international in Western Kenya (the INULA study)[27],with a 15% desired increase in the proportion at the end of the project, at 95% confidence level, 95% power and a design effect of 2. Ten sublocations were randomly sampled from the list of sublocations in Vihiga County proportionally according to number of households living in each sublocation. Within each selected sublocation and with the help of community health volunteers (CHVs) and the local administration (assistant chiefs and village elders), a list of all households with a child between 12 and 23 months was composed. Subsequently, forty households were randomly sampled from each list for a total sample size of 400 per survey round. All households sampled in the plenty season that still met the sampling criteria (n = 151) (child between 12 and 23 months) were included in the lean season sample and the sample was refilled with randomly selected households from a new list of households meeting the inclusion criteria at the time of the second survey round. In the end, 249 new households were sampled in the lean season in addition to the 151 households from the plenty season.

### Data collection

The dietary intake data were collected by enumerators with a background in nutrition while the agricultural data were collected by enumerators with a background in agricultural studies. All enumerators were trained and the data collection tools pre-tested following standardized procedures.

#### Agrobiodiversity data

Information about household on-farm biodiversity was collected once for each household including the 151 households sampled twice. An interviewer administered semi–structured questionnaire was used to gather the information from the household head or spouse. The respondent was asked to draw a sketch of the farms, plots or kitchen gardens owned by the household. Subsequently, for each land type a list with all useful plant species grown during the long and short rainy seasons as well as any other useful (semi-)wild species was composed. Equally, a list of all useful animal species maintained by the household on their farm was composed. A useful plant species was defined as a plant species used as food, animal feed, medicine, fuel, mulch or construction material and a useful animal species defined as edible animal species maintained by the household for income, food, fuel or manure. This was followed by farm visits and forest walks by trained technicians to collect specimens for all the plant species mentioned in both survey rounds following standard collection procedures [28] followed by identification at the Botany department of National of Museums of Kenya.

#### Dietary data

Except for the 151 households that had dietary data collected twice in the lean and plenty seasons, dietary data for the rest of the sample was collected once using a quantitative 24-hour dietary intake recall repeated twice on non-consecutive days following the methodology described by Gibson and Ferguson [29]. For the purpose of this study, the data from the plenty season was utilized for the households that had the dietary data collected in both seasons. The respondents were mothers or primary caregivers who were responsible for food preparation and feeding of the children. The respondent was asked to describe all the foods and beverages consumed including those eaten away from home by the children during the day previous to the interview (24-hour period). The quantities cooked and eaten were estimated using household measures such as cups, spoons, and bowls; molding clay, water, market prices and where available direct weighing of the foods was done. Weights of ingredients consumed were estimated in raw forms and expressed as proportion of the total weights of food prepared in order to estimate the exact quantities of the food or ingredients consumed. For foods consumed or prepared outside the home, standard recipes were calculated.

The amounts of foods and ingredients consumed entered into the Lucille software [30] for conversion into nutrients intakes for each child. For this purpose a food composition table for the area was composed based primarily on the Tanzanian food composition table [31] and uploaded in Lucille. Missing foods and nutrients were supplemented with values from the Kenyan food composition table[32], the USDA table [33]and the West African tables [34]. The Kenyan table was not used as the primary table because it has many missing foods and it is relatively old. The values were corrected for nutrient retention using the USDA table of nutrient retention factors, release 6 [35]. The nutrient intakes from the two recalls were converted to usual intakes using the Multiple Source Method (MSN) program [36].

### Data management and statistical analysis

#### Household socio-economic index (SEI)

The principal component analysis (PCA) was used to construct a household SEI in SPSS using variables on asset ownership, sanitation facilities and housing characteristics of the main house [37]. Using the index, households were grouped into five economic quintiles: poorest, poor, medium, wealthy and wealthiest.

#### Household agrobiodiversity

The household on–farm agrobiodiversity (household ABD) was assessed using Crop species Richness (CSR) and Livestock Species Richness (LSR) as recommended by the publication by Last et al [38]. CSR is the total number of wild or cultivated plant species for agricultural purpose per farm while LSR is the total number of livestock species occurring per farm. The household ABD score is the sum of CSR and LSR.

#### Dietary micronutrient quality

The quality of children’s micronutrient intake was assessed using two indicators: The dietary diversity score (DDS), the probability of adequate intake (PA) and the mean probability of adequacy (MPA). Research has shown that DDS and food variety scores based on a count of food item consumed are good proxies of nutrient adequacy [39–43]. However the mean adequacy ratio (MAR) and the MPA remain gold standard in estimating micronutrient adequacy [39, 41].

The DDS was calculated as the sum of the number of food groups consumed by the child in the 24 hours preceding the interview day. The first recall was used to compute the DDS and MDDS from the 24-h recall data. Seven food groups (Grains, roots and tubers; Legumes and nuts; Dairy products; Flesh foods; Eggs; Vitamin A rich fruits and vegetables; and Other fruits and vegetables) were used to compute the score [44]. The MDDS was calculated as the proportion of children with DDS of 4 or more.

The PA was calculated for the following 11 micronutrients vitamin A, vitamin C, thiamin, riboflavin, niacin, vitamin B6, vitamin B12, folate, calcium, iron and zinc. The PA for all the micronutrients except iron was assessed from the respective Estimated Average Requirements (EAR) and standard deviations (SD) using the CDF.NORMAL function of SPSS following the Institute of Medicine guidelines [45]. The EAR values were those published by FAO/WHO while the SD values were derived from coefficients of variation (CV) of the respective micronutrients [46]. Due to the skewed nature of the distribution of the requirements for iron in children, the full probability approach was used to estimate probability of adequacy using values [29, 46]. The bioavailability of iron and zinc is affected by the composition of the diet. We used the low–bioavailability values for zinc and 5% bioavailability for iron. The MPA, an overall measure of micronutrient adequacy of the diet, was calculated as an average of the individual nutrient PAs and expressed as a percentage. The population prevalence of adequacy is the average of the individual probabilities of adequacy.

All the statistical analyses were done using IBM SPSS statistics version 22. Descriptive statistics were computed for the categorical variables using percentages and for continuous data using means, standard deviations, medians, minimums and maximum. Group differences of categorical and continuous variables were explored using Chi- square tests and t–tests respectively. Bivariate regression analysis was used to explore the correlation between the dependent variables DDS, PA, MPA and the household ABD score (CSR and LSR) and other independent variables. The Hierarchical multivariate regression analysis was used to estimate the relationship between agrobiodiversity and the children’s diet.

### Ethical considerations

The study was approved by the Ethics and Review Committee of Egerton University (REF: EU/DVCRE/009). Written consent was obtained once from all respondents at the first visits and an oral consent in the subsequent visits.

## Results

### Demographics

Of the 649 unique households sampled (400 in the plenty season and 249 in the lean season) for the plenty and lean seasons fifteen (15) households were excluded from the analysis due to incompleteness of the data. The final pooled sample comprised 634 unique households. Table 1 presents the characteristics of the pooled sample. Only 13.4% of the respondents for the agrobiodiversity questionnaire were males. The mean age of the respondents for the agrobiodiversity questionnaire was 34±12.9 years. Fourteen children included in the sample were older than the target age (24≤age ≤31 months) while four were slightly younger (10≤age ≤11 months). The mean age of the household heads was 41.2±13.7 years. Of the households, 84.2% were male headed and 95.3% of them were in monogamous type of marriage. According to the household wealth index, 14.8% of the households were classified as poorest and another 25.1% as poor. The primary female caregivers had a mean age of 30.3±10.5 years.

**Table 1:** Socio-demographics of the sample.

Characteristic	Percent, N = 634
Gender of household head, male	84.2
Marital profile of household, monogamy	95.3
Age of the household head in years, (mean ±SD (min, max))	41.2±13.7(20, 90)
*Education level of Household head*	
None	2.7
Primary, incomplete	32.5
Primary, completed	34.3
Secondary, incomplete	7.0
Secondary, completed	17.6
Tertiary	6.0
*Monthly income*	
Less than Ksh. 3500	36.7
Ksh. 3500–7000	44.3
Ksh. 7000–14000	10.7
More than Ksh. 14000	8.3
*Household SEI ranking*	
Poorest	14.8
Poor	25.1
Medium	20.0
Wealthy	20.0
Wealthiest	20.0
Caregiver's age, (mean ± SD (min, max))	30.2±10.2 (17, 74)
*Caregiver's education*	
None	4.3
Primary, incomplete	33.2
Primary, completed	35.7
Secondary, incomplete	11.2
Secondary, completed	11.5
Tertiary	4.1
Sex of child, male	50.0
Age of the child in months, (mean ±SD (min, max))	18.1±3.8 (10.3, 31.1)

### On farm agrobiodiversity score

**Fig 1:**
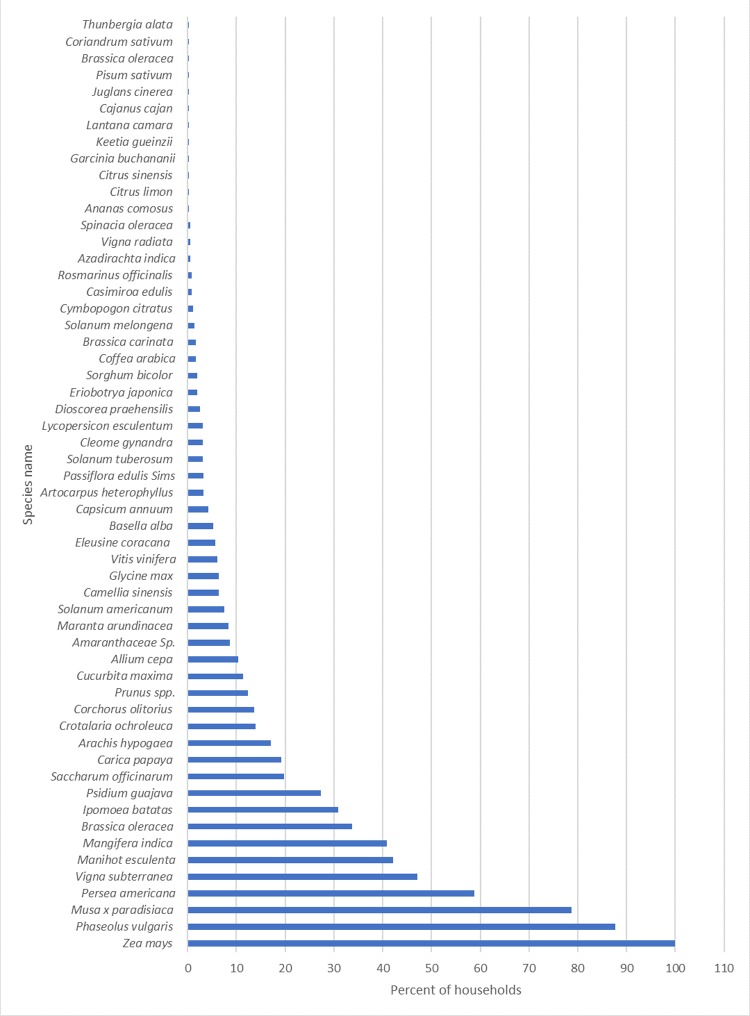
Household distribution of edible crop species on farms.

A total of 80 different edible on farm species (67 plant species and 13 animals’ species) were listed by the households in the sample. The mean household ABD score was 9.9±4.3 (median = 9; min = 1; max = 27). The means for the CSR and LSR were 8.2±3.8 (min = 1, max = 24) and 1.8±1.0 (min = 0, max = 6) respectively. About 20.4% of the households did not own any livestock. Of the crop species listed, 20 were used as vegetables, 19 as fruits, 12 as pulses, legumes and nuts, 5 as roots and tubers, 4 as cereals, 2 as beverages, 2 as condiments, 1 banana, 1 as high sugar crop (sugarcane) and another 1 as infusion. The age of the household head was positively correlated with the CSR (r = 0.24, p<0.000), LSR (r = 0.27, p<0.000) and the household ABD score (r = 0.29, p<0.000). The female headed households had significantly higher means for CSR than the male headed households (9.17±4.34 vs 7.98±3.7, t_619_ = -2.863, p = 0.004). The difference in means for the LSR however did not reach significant levels between the male headed households (1.76±1.01) and female headed household (1.76±0.88; t _630_ = -0.006, p = 0.996). A correlation analysis between the household ABD indicators and household SEI found a positive significant correlation with LSR (r = 0.16, p<0.000) but not with CSR (p>0.05). Fig 1 describes the proportions of households growing the various crop species on their farms. Just under two-thirds (65.7%) of the species listed were grown by less than 10% of the households. Only five species were grown by more than half of the households. These were: Maize (*Zea mays* L.) in 96.1%, Beans (*Phaseolus vulgaris* L.) in 88.9%, Banana (*Musa x paradisiaca* L.) in 75.5%, avocado (*Persea americana* Mill.) in 61.7% and Cowpeas (*Vigna unguiculata* (L.) in 53.1% of the households. On the other hand, chicken, cattle and goats were the most popular animals reared in 73.5%, 53.8% and 15.9% of the households respectively. All the other animal species were listed in less than 3% of the households surveyed.

### Children’s dietary diversity

The children’s DDS ranged from 1 to 6 in both seasons with statistically different means in the two survey rounds of 4.2±1.04 in the plenty season and 3.9±0.98 in the lean season (t _(628)_ = 2.487, p = 0.013). About 22.8% of the children did not meet the minimum recommended dietary diversity score of 4 or more food groups in the plenty season compared with 27.6% children in the lean season. This difference in proportions did not reach a statistically significant level (χ^2^ (1, N = 630) = 1.883, p = .17). Nearly all the children consumed foods from the grains, roots and tubers group. This was followed by the vitamin A rich fruits and vegetables group, dairy products, and other fruits and vegetables. Other than the dairy products group, consumption of animal source foods was very low. Table 2 describes the proportion of children consuming foods from the different food groups in the two survey rounds. The consumption of foods in the legumes and nuts, dairy products and eggs were significantly lower in the lean season than in the plenty season. A correlation analysis between the DDS and the socio-demographic characteristics showed a positive significant correlation between DDS and household SEI (r = 0.19, p<0.000), educational level of household head (r = 0.14, p<0.001), caregiver’s educational levels (r = 0.12, p<0.001) and child’s age (r = 0.15, p<0.000).

**Table 2:** Proportions of children consuming foods from different food groups in the two seasons.

Food group	Plenty season (%)	Lean season (%)	Significance (n = 630)
Grains, roots and tubers	99.5	99.6	0.869
Legumes and nuts	23.0	13.4	0.003
Dairy products	85.2	78.7	0.036
Flesh foods	31.5	34.3	0.458
Eggs	3.1	0.4	0.023
Vitamin A rich fruits and vegetables	90.5	88.3	0.367
Other fruits and vegetables	81.8	79.1	0.393

### Probability of adequacy of micronutrients

The children’s mean caloric intake in the plenty season was 1210.59±391.09 (median = 1164.91) kcals compared to 1224.30±479.45 median = 1179.48) kcals (t_632_ = -.393, p = .695). The children’s usual caloric intake was positively associated with the household SEI in both the plenty season (r = 0.22, p<0.000) and in the lean season (r = 0.21, p = 001). The children’s micronutrient requirement, intakes and probability of adequacies are described in Table 3. The MPA of the children ranged from 9% to 99% (median = 67.0%) in the plenty season and 0% to 98% (median = 57.5%) in the lean season. About 13.4% of the children had low MPA (<50%) during the plenty season compared to 32.2% in the lean season, slightly more than half of the children (51.3% in the plenty season versus 52.3% in the lean season) had adequacy levels of 50–75% and the rest of the children, (35.3% in the plenty season versus 15.5% in the lean season) had high adequacy levels (≥75%). The difference in proportions reached significant levels for the <50% and the ≥75% groupings across the two seasons (χ^2^ (2, N = 627) = 46.431, p = .000). Overall, the prevalence of nutrient adequacy was 68.11% during the plenty season compared to 56.37% in the lean season (t_629_ = 8.201, p = 0.000) and was positively correlated with the DDS both in the plenty season (r = 0.42, p<0.00) and in the lean season (r = 0.53, p = 0.000).

**Table 3:** Children’s micronutrient requirements, intakes and prevalence of adequacy.

	Requirements	Intakes			Prevalence of micronutrient adequacy, %		
	EAR, (SD)	Mean ±SD, (Median)			Mean ± SD		
		Plenty season	Lean Season	*P*	Plenty season	Lean season	*p*
Vitamin A (µgRE)	286, (57)	608.71 ± 377.83, (538.54)	218.66± 114.52, (194.23)	0.000*	80.36 ± 34	24.21 ± 33.97	0.000*
Vitamin C (mg)	25, (2.5)	83.15 ± 47.98, (72.5)	62.48± 41.4, (52.63)	0.000*	91.89 ± 25.17	86.07 ± 32.6	0.012*
Thiamin (mg)	0.4, (0.05)	0.73 ± 0.25, (0.7)	0.68± 0.28, (0.66)	0.026*	92.67 ± 21.81	85.14 ± 31.72	0.000*
Riboflavin (mg)	0.4, (0.05)	2.48 ± 1.63, (1.9)	1.3± 0.64, (1.14)	0.000*	99.81 ± 3	97.7 ± 14.34	0.005*
Niacin (mg)	5, (0.5)	7.36 ± 2.56, (7)	7.49± 3.1, (7.19)	0.568	83 ± 32.01	79.19 ± 36.59	0.169
Vitamin B6 (mg)	0.4, (0.05)	1.1 ± 0.51, (1.01)	0.84± 0.38, (0.78)	0.000*	96.46 ± 15.41	91.25 ± 24.03	0.001*
Folate (g)	120, (15)	166.12 ± 70.42, (153.59)	99.7± 41.14, (92.69)	0.000*	70.92 ± 39.35	28.09 ± 38.5	0.000*
Vitamin B12 (g)	0.7, (0.1)	1.08 ± 0.81, (0.93)	1.12± 0.7, (0.95)	0.563	64.29 ± 44.27	69.41 ± 41.24	0.147
Calcium (mg)	417, (41.5)	342.39 ± 199.85, (308.51)	303.54± 186.32, (259.35)	0.015*	14.92 ± 28.92	13.11 ± 27.7	0.436
Iron (mg)	-	7.98 ± 2.77, (7.58)	7.55± 3.2, (7.22)	0.075	27.39 ± 39.7	21.46 ± 38.34	0.065
Zinc (mg)	6.9, (0.7)	5.05 ± 1.68, (4.77)	4.75± 1.87, (4.56)	0.037*	27.95 ± 19.1	25.63 ± 20.72	0.151
MPA	-				68.11 ± 16.08	56.37 ± 19.5	0.000*

*Significant *p*-values

The MPA was positively and significantly correlated with the following sociodemographic variables: household SEI (r = 0.22, p<0.000), age of the household head (r = 0.1, p<0.015), educational level of the household head (r = 0.13, p < 0.001), caregiver’s educational level (r = 0.17, p<0.000), caregiver’s age (r = 0.11, p<0.007), and age of the child (r = 0.16, p<0.000).

The PA for all the three minerals were below 30% with zinc being the most limiting micronutrient followed by calcium and iron. Folate was the most limiting vitamin followed by vitamin A. Practically all the children meet their requirements for riboflavin.

### Association between children’s dietary quality and household on farm agrobiodiversity

A bivariate regression analysis with the pooled sample showed no significant correlation between the household ABD score and children’s caloric intakes (p>0.05). There was however a significant, mild positive association between the household ABD score and DDS (r = 0.09, p = 0.029) as well as with MPA (r = 0.15, p<0.000) for the pooled sample. Hierarchical multiple linear regression analysis was used to examine the predictors of DDS and MPA. In the first block, the demographic variables were entered (sex of the household head, age of household head, education of household head, household SEI, education of caregiver, age of caregiver and the sex and age of the child) and in the second block the household ABD score was entered. In predicting DDS, significant model emerged for the demographics, *F* (8, 625) = 6.05, p<0.001, R^2^ = 0.074. However, inclusion of household ABD score did not yield significant change to the model: *ΔF* (1, 624) = 3.44, *Δp*>0.05, *ΔR*^*2*^ = 0.005. As shown in Table 4, only the household SEI and child’s age were significant predictors of child DDS. In predicting MPA, the first model for demographics was significant: *F* (8, 625) = 9.13, p<0.000, R^2^ = 0.105. Inclusion of household ABD score in the prediction and controlling for the sociodemographic variables yielded a significant change in the prediction: *ΔF* (1, 624) = 10.451, *Δp*<0.001, *ΔR*^*2*^ = 0.015. The results of the full model are presented in Table 5. The results show that, a one unit increase in the number of species on the farm was associated with 12.7% increase in the MPA. The household SEI (ß = .17, t (625) = 3.986, p<0.000), mother’s educational levels (ß = .13, t (625) = 3.0, p<0.003), caregiver’s age (ß = .082, t (625) = 1.984, p<0.048), child age (ß = .163, t (629) = 4.304, p<0.000) were the significant socio-demographic predictors of MPA. Further analysis was conducted to determine which between the CSR and LSR was predictive of MPA. The model showed that CSR was a significant predictor of MPA (ß = .139, t (631) = 3.366, p<0.001). The LSR was however not predictive of MPA (ß = .028, t (631) = 0.690, p<0.490).

**Table 4:** Results of the final model of the multiple regression analysis for the prediction of DDS.

	ß	t	Sig.	95% CI for ß		
				Lower	Upper	*pr*^*2*^
Household SEI	0.146	3.413	**0.001**	0.064	0.236	0.018
Gender of household head (1 = male, 2 = female)	0.034	0.840	0.401	-0.127	0.315	0.001
Educational level of household head	0.049	1.059	0.290	-0.033	0.111	0.002
Age of household head (years)	0.021	0.481	0.631	0.005	0.008	0.004
Caregivers age in years	-0.037	-0.885	0.377	-0.012	0.005	0.003
Educational level of caregiver	0.058	1.315	0.189	-0.024	0.123	0.003
Sex of the child (1 = male, 2 = female)	-0.063	-1.637	0.102	-0.285	0.026	0.004
Age of the child (months)	0.162	3.413	**0.001**	0.023	0.064	0.027
Household overall ABD score	0.075	1.855	0.064	-0.001	0.036	0.005

**Table 5:** Results of the final model of the multiple regression analysis for the prediction of MPA.

	ß	t	Sig.	95% CI for ß		
				Lower	Upper	*pr*^*2*^
Household SEI	0.167	3.922	**0.000**	0.015	0.046	0.025
Gender of household head (1 = male, 2 = female)	0.056	1.416	0.157	-0.011	0.068	0.003
Educational level of household head	0.006	0.130	0.896	-0.012	0.014	0.000
Age of household head (years)	0.010	0.218	0.828	-0.001	0.001	0.000
Caregivers age in years	0.082	1.945	0.052	0.000	0.003	0.006
Educational level of caregiver	0.130	2.976	**0.003**	0.007	0.033	0.014
Sex of the child (1 = male, 2 = female)	-0.046	-1.195	0.233	-0.045	0.011	0.002
Age of the child (months)	0.165	4.287	**0.000**	0.004	0.012	0.030
Household overall ABD score	0.127	3.172	**0.002**	0.002	0.009	0.016

## Discussion

The purpose of this article was to explore the association between the household on-farm agrobiodiversity and dietary micronutrient adequacy. As no single food contains all necessary nutrients, diversity in dietary sources is needed to ensure a balanced and healthy diet. We therefore measured micronutrient adequacy using two indicators, the DDS and MPA. The two were shown to be highly correlated by other researchers [39–41] as is the case with the current study. Our analysis of the dietary diversity revealed that the diets of the children was not highly diversified. The mean DDS of the children was 4.0 which coincides with recommended cut-off for the minimum dietary diversity [44]. About one in every four children did not meet this minimum recommended DDS. This can be linked to the lack low agrobiodiversity in most households despite the high agrobiodiversity within the general community.

The choice of what is consumed by the community is determined by many factors including what is available in the market and on-farm. The current study found a positive significant correlation between the two indicators of micronutrient adequacy and the household on-farm agrobiodiversity. The strength of the association in our study between DDS and household agrobiodiversity was however very small and, in further analysis, did not significantly change the prediction model using hierarchical regression analysis. This was however not expected given that in this study we have documented a high number of edible plant and animal species painting the study area as a highly biodiverse area with several options for diversifying diets of the communities. Nonetheless, taking into account our study finding that shows a diminishing trend in CSR where more than 65% of the species are cultivated by less than 10% of the farmers, this is not a surprise as most households had low agrobiodiversity that could contribute to dietary diversity. This study and others [16]have recorded dominance of maize on farm that could be linked to the dominance of diet of the children by maize based foods. On the other side, one study with a one week recall period reported that agrobiodiversity was responsible for close to 48.5% of the variation in the diets of children studied [4]. The lack of significant association between DDS and ABD in our study could also be due to the short recall period different from the seven day period in the study by Ekesa et al [4].

The overall prevalence of micronutrient inadequacy was 36.4%. Only 28% of the children had high overall adequacy levels (≥75%) while 20% did not meet half of their overall dietary requirements. The average probability of adequacy fell below 75% for 6 of the 11 micronutrients considered. Minerals were the most limiting micronutrients with average PAs below 30%. Our results corroborate those of a recent study conducted in Kitui and Vihiga counties that identified similar to our study iron, zinc and calcium as the problem micronutrients. The study defined problem nutrients as those nutrients that cannot reach 100% of the recommended nutrient intakes (RNI) in the nutritionally best possible diets when modelled in Optifood software [47]. In Vihiga, among children between 12 and 23 months old, the micronutrients calcium, iron and zinc only reached 86, 60, and 61% respectively of the RNI. The current study also agrees with those of national surveys. The latest national micronutrient survey [48] indicated that 42.3 and 34.6% of the children 12 -23months of age are suffering from anemia and iron deficiency anemia respectively. The same survey reported low plasma zinc levels in 85.3% of the children. Our results therefore points to poor dietary diversification and nutrient intakes as a possible cause of the nutrient inadequacies among other causes and conforms with the findings of the national health survey reporting that only 41% of children 12 – 23months old ate foods rich in iron within a 24-hour recall period [3].

In the prediction of MPA, household agrobiodiversity contributed significantly to the model. The model shows that increment of household agrobiodiversity by one species was associated with an increase in the children’s probability of nutrient adequacy by 12 percentage points. Discriminant analysis further showed that the number of livestock species maintained by the household did not affect the diet of the children significantly. In the current study close to 80% of the households reared animals but only about one-third (32.5%) of the children consumed animals source foods (apart from the dairy products). Consumption of foods of animal origin was very low with the exception of the dairy products group. About 12% of the children did not consume any food of animal origin during the recall period. Though many children consumed milk, it may not have contributed significantly to the overall quality of the diet since the milk was often taken as tea with very small quantities of milk diluted in large quantities of water. Despite 92.2% of the households reporting rearing chicken only 2.1% of the children consumed eggs during the recall period. Our findings affirm earlier studies that reported minimal consumption of animal source foods and diets low in variety and diversity among children in western Kenya [4], [49], [50] and even nationally [48]. Ekesa et al [4] reported that up to 11% of the children did not consume any food of animal origin within a one-week recall period. Other studies linking livestock ownership and child growth in children in western Kenya showed lack of association between owning higher numbers of household livestock and child measures or subsequent child linear growth outcomes[51].This implies that owning more animals might not directly translate in increased consumption of nutritionally rich foods.

Our study has some limitations. First, relying on the recall of a single household member to determine agrobiodiversity of the entire household is likely to underestimate the total agrobiodiversity of the households especially in the households where men and women own or oversee crops for different purposes. Secondly, the use of one survey dietary data for association with the entire agrobiodiversity for two seasons can also lead to underestimation of the contribution of the agrobiodiversity to the diets. Lastly, asking the respondents to recall the crops grown and wild plants harvested the previous season can also lead to underestimation of the actual household agrobiodiversity.

## Conclusions

Our study contributes significantly to the growing body of knowledge linking agrobiodiversity to nutrition of smallholder farmers. The study shows that Vihiga County is very rich in agrobiodiversity. However, this rich diversity is diminishing due to reliance on a few species by many farmers leaving many other–nutritious—species underutilized. Despite the rich diversity, the diets are low in diversity and micronutrient content with very limited consumption of animal source foods. Policy makers and program implementers aiming to improve diets of communities living in rural areas should aim at promotion of utilization of agrobiodiversity innovatively. For example, integrating local, neglected and underutilized plant species in home gardening and promoting their access through community actions such as biodiversity fairs, diversity kits and establishing community-based home garden resource centers and use of community platforms such as breastfeeding mothers’ clubs and merry-go rounds to promote local agrobiodiversity. Other avenues of promotion of the underutilized nutritious species can be through schools either through learning plots or through school meals programs.

## References

[1] Thompson B., Amoroso L., C.A.B. Eds.; Improving Diets and Nutrition: Food-Based Approaches. Food and Agriculture Organization of the United Nations and CABI; Food and Agriculture Organization of the United Nations: Wallingford, Oxfordshire: Rome, Italy, 2014.

[2] Victora C. G.; Adair L.; Fall C.; Hallal P. C.; Martorell R.; Richter L.; Sachdev H. S.; Maternal and Child Undernutrition Study Group. Maternal and Child Undernutrition: Consequences for Adult Health and Human Capital. Lancet Lond. Engl. 2008, 371 (9609), 340–357. 10.1016/S0140-6736(07)61692-4 PMC2258311 18206223

[3] Kenya National Bureau of Statistics; Ministry of Health/Kenya; National AIDS Control Council/Kenya; Kenya Medical Research Institute; National Council for Population and Development/Kenya; ICF International. Kenya Demographic and Health Survey 2014; Rockville, MD, USA, 2015.

[4] Ekesa B. N.; Walingo M. K.; Abukutsa-Onyango M. O. Influence of Agricultural Biodiversity on Dietary Diversity of Preschool Children in Matungu Division, Western Kenya. Afr. J. Food Agric. Nutr. Dev. 2008, 8 (4), 390–404.

[5] Ng’endo M.; Bhagwat S.; Keding G. B. Contribution of Nutrient Diversity and Food Perceptions to Food and Nutrition Security Among Smallholder Farming Households in Western Kenya: A Case Study. Food Nutr. Bull. 2017, 0379572117723135. 10.1177/0379572117723135 28826252

[6] Dioula, B. M.; Deret, H.; Morel, J.; Kiaya, V. Enhancing the Role of Smallholder Farmers in Achieving Sustainable Food and Nutrition Security.

[7] Kahane R.; Hodgkin T.; Jaenicke H.; Hoogendoorn C.; Hermann M.; Keatinge J. D. H. (Dyno); et al Agrobiodiversity for Food Security, Health and Income. Agron. Sustain. Dev. 2013, 33 (4), 671–693. 10.1007/s13593-013-0147-8

[8] Powell B.; Thilsted S. H.; Ickowitz A.; Termote C.; Sunderland T.; Herforth A. Improving Diets with Wild and Cultivated Biodiversity from across the Landscape. Food Secur. 2015 10.1007/s12571-015-0466-5

[9] Keding, G. B.; Cogill, B. Linking Nutrition and Agrobiodiversity; Food and Agriculture Organization of the United Nations, 2013.

[10] Frei M.; Becker K. Agro-Biodiversity in Subsistence-Oriented Farming Systems in a Philippine Upland Region: Nutritional Considerations. Biodivers. Conserv. 2004, 13 (8), 1591–1610. 10.1023/B:BIOC.0000021330.81998.bb

[11] Food and Agriculture Organization; Platform for Agrobiodiversity Research. Biodiversity for Food and Agriculture: Contributing to Food Security and Sustainability in a Changing World; 2011.

[12] FAO; IFAD; WFP. The State of Food Insecurity in the World: Meeting the 2015 International Hunger Targets: Taking Stock of Uneven Progress.; 2015. https://doi.org/I4646E/1/05.15 10.3945/an.115.009936 PMC4561839 27352453

[13] Khoury C. K.; Bjorkman A. D.; Dempewolf H.; Ramirez-Villegas J.; Guarino L.; Jarvis A.; et al Increasing Homogeneity in Global Food Supplies and the Implications for Food Security. Proc. Natl. Acad. Sci. 2014, 111 (11), 4001–4006. 10.1073/pnas.1313490111 24591623 PMC3964121

[14] Jones A. D.; Shrinivas A.; Bezner-Kerr R. Farm Production Diversity Is Associated with Greater Household Dietary Diversity in Malawi: Findings from Nationally Representative Data. Food Policy 2014, 46, 1–12. 10.1016/j.foodpol.2014.02.001

[15] Hillocks R. J. Farming for Balanced Nutrition: An Agricultural Approach to Addressing Micronutrient Deficiency among the Vulnerable Poor in Africa. Afr. J. Food Agric. Nutr. Dev. 2011, 11 (2).

[16] Ng’endo M.; Keding G. B.; Bhagwat S.; Kehlenbeck K. Variability of On-Farm Food Plant Diversity and Its Contribution to Food Security: A Case Study of Smallholder Farming Households in Western Kenya. Agroecol. Sustain. Food Syst. 2015, 39 (10), 1071–1103. 10.1080/21683565.2015.1073206

[17] Mburu S. W.; Koskey G.; Kimiti J. M.; Ombori O.; Maingi J. M.; Njeru E. M. Agrobiodiversity Conservation Enhances Food Security in Subsistence-Based Farming Systems of Eastern Kenya. Agric. Food Secur. 2016, 5 (1), 19 10.1186/s40066-016-0068-2

[18] Bahadur KC K.; Pant L. P.; Fraser E. D. G.; Shrestha P. K.; Shrestha D.; Lama A. Assessing Links between Crop Diversity and Food Self-Sufficiency in Three Agroecological Regions of Nepal. Reg. Environ. Change 2016, 16 (5), 1239–1251. 10.1007/s10113-015-0851-9

[19] Walingo M. K.; Ekesa B. N. Nutrient Intake, Morbidity and Nutritional Status of Preschool Children Are Influenced by Agricultural and Dietary Diversity in Western Kenya. Pak. J. Nutr. 2013, 12 (9).

[20] Hirvonen K.; Hoddinott J. Agricultural Production and Children’s Diets: Evidence from Rural Ethiopia. Agric. Econ. 2017, 48 (4), 469–480. 10.1111/agec.12348

[21] Jones A.D.; Creed-Kanashiro H.; Zimmerer K.S.; de Haan S.; Carrasco M.; Meza K.; et al Farm-Level Agricultural Biodiversity in the Peruvian Andes Is Associated with Greater Odds of Women Achieving a Minimally Diverse and Micronutrient Adequate Diet, The Journal of Nutrition, 2018, 148(10), 1625–1637, 10.1093/jn/nxy166 30219889

[22] Vihiga County Intergrated Development Plan (2013–2017) | Millennium Development Goals | Kenya https://www.scribd.com/document/202779259/Vihiga-County-Intergrated-Development-Plan-2013-2017 (accessed Oct 5, 2017).

[23] Kindt R.; Van Damme P.; Simons A. J. Patterns of Species Richness at Varying Scales in Western Kenya: Planning for Agroecosystem Diversification. Biodivers. Conserv. 2006, 15 (10), 3235–3249. 10.1007/s10531-005-0311-9

[24] The Kenya National Bureau of Statistics. County Statistics. Kenya National Bureau of Statistics, 2013.

[25] Kenya National Bureau of Statistics. Exploring Kenya’s Inequality: Pulling apart or pooling together? Vihiga county http://inequalities.sidint.net/kenya/wp-content/uploads/sites/2/2013/09/Vihiga.pdf (accessed Oct 6, 2017).

[26] Magnani, R. Sampling Guide; Food Security and Nutrition Monitoring (IMPACT) Project, 1999.

[27] Waswa, L.M. Improving Dietary Diversity and Nutritional Health of Women and Children under Two Years through Increased Utilization of Local Agrobiodiversity and Enhanced Nutrition Knowledge in Kenya. PhD Dissertation, Justus-Liebig-University Giessen, Germany. 2016.

[28] Bridson D.; Forman L. The Herbarium Handbook; Royal Botanic Gardens, 1998.

[29] Gibson, R. S.; Ferguson, E. L. An Interactive 24-Hour Recall for Assessing the Adequacy of Iron and Zinc Intakes in Developing Countries. 2008.

[30] Lucille: software to process your food intake data | Department of Food Safety and Food Quality http://www.foodscience.ugent.be/nutriFOODchem/foodintake (accessed Sep 30, 2017).

[31] Lukmanji, Z.; Hertzmark, E.; Mlingi, N.; Assey, V.; Ndossi, G.; Fawzi, W. Tanzania Food Composition Tables. MUHAS-TFNC HSPH Dar Es Salaam Tanzan. 2008.

[32] Sehmi J. K. National Food Composition Tables and The Planning of Satisfactory Diets in Kenya; Government Printer: Nairobi, Kenya, 1993.

[33] U.S. Department of Agriculture, Agricultural Research Service. USDA National Nutrient Database for Standard Reference, Release 27. Nutrient Data Laboratory Home Page. 2014.

[34] Stadlmayr, B.; Food and Agriculture Organization of the United Nations; International Network of Food Data Systems; Economic Community of West African States; Bioversity International. West African Food Composition Table = Table de Composition Des Aliments d’Afrique de l’Ouest; 2012

[35] U.S. Department of Agriculture. Nutrient Data Laboratory—2007—USDA Table of Nutrient Retention Factors, Release 6. 2007.

[36] MSM https://msm.dife.de/ (accessed Sep 30, 2017).

[37] Vyas S.; Kumaranayake L. Constructing Socio-Economic Status Indices: How to Use Principal Components Analysis. Health Policy Plan. 2006, 21 (6), 459–468. 10.1093/heapol/czl029 17030551

[38] Powell B.; Thilsted S. H.; Ickowitz A.; Termote C.; Sunderland T.; Herforth A. Improving Diets with Wild and Cultivated Biodiversity from across the Landscape. Food Secur. 2015 10.1007/s12571-015-0466-5

[39] Kennedy G. L.; Pedro M. R.; Seghieri C.; Nantel G.; Brouwer I. Dietary Diversity Score Is a Useful Indicator of Micronutrient Intake in Non-Breast-Feeding Filipino Children. J. Nutr. 2007, 137 (2), 472–477. 10.1093/jn/137.2.472 17237329

[40] Daniels M. C.; Adair L. S.; Popkin B. M.; Truong Y. K. Dietary Diversity Scores Can Be Improved through the Use of Portion Requirements: An Analysis in Young Filipino Children. Eur. J. Clin. Nutr. 2009, 63 (2), 199–208. 10.1038/sj.ejcn.1602927 17971828

[41] Rathnayake K. M.; Madushani P. A. E.; Silva K. Use of Dietary Diversity Score as a Proxy Indicator of Nutrient Adequacy of Rural Elderly People in Sri Lanka. BMC Res. Notes 2012, 5 (1), 469. 22931957 10.1186/1756-0500-5-469 PMC3470944

[42] Gewa C. A.; Murphy S. P.; Weiss R. E.; Neumann C. G. Determining Minimum Food Intake Amounts for Diet Diversity Scores to Maximize Associations with Nutrient Adequacy: An Analysis of Schoolchildren’s Diets in Rural Kenya. Public Health Nutr. 2014, 17 (12), 2667–2673. 10.1017/S1368980014000469 24690343 PMC10282467

[43] Mirmiran P.; Azadbakht L.; Azizi F. Dietary Diversity within Food Groups: An Indicator of Specific Nutrient Adequacy in Tehranian Women. J. Am. Coll. Nutr. 2006, 25 (4), 354–361. 16943458 10.1080/07315724.2006.10719546

[44] World Health Organization. Indicators for Assessing Infant and Young Child Feeding Practices: Part 2: Measurement. 2010.

[45] Institute of Medicine (US) Subcommittee on Interpretation and Uses of Dietary Reference Intakes; Institute of Medicine (US) Standing Committee on the Scientific Evaluation of Dietary Reference Intakes. DRI Dietary Reference Intakes: Applications in Dietary Assessment; National Academies Press (US): Washington (DC), 2000. 25057725

[46] Allen L. Guidelines on Food Fortification with Micronutrients; World Health Organization: Food and Agriculture Organization of the United Nations: Geneva, 2006.

[47] Daelmans B.; Ferguson E.; Lutter C. K.; Singh N.; Pachón H.; Creed-Kanashiro H.; et al Designing Appropriate Complementary Feeding Recommendations: Tools for Programmatic Action. Matern. Child. Nutr. 2013, 9, 116–130. 10.1111/mcn.12083 24074322 PMC6860844

[48] Kenya National Bureau of Statistics (KNBS); Division of Nutrition, Ministry of Public Health and Sanitation (MOPHS); Kenya Medical Research Institute (KEMRI). The Kenya National Micronutrients Survey 2011; 2011.

[49] Walingo M. K.; Ekesa B. N. Nutrient Intake, Morbidity and Nutritional Status of Preschool Children Are Influenced by Agricultural and Dietary Diversity in Western Kenya. Pak. J. Nutr. 2013, 12 (9), 854–859. 10.3923/pjn.2013.854.859

[50] Ferguson E.; Chege P.; Kimiywe J.; Wiesmann D.; Hotz C. Zinc, Iron and Calcium Are Major Limiting Nutrients in the Complementary Diets of Rural Kenyan Children. Matern. Child. Nutr. 2015, 11 Suppl 3, 6–20. 10.1111/mcn.12243 26778799 PMC5066654

[51] Mosites E. M.; Rabinowitz P. M.; Thumbi S. M.; Montgomery J. M.; Palmer G. H.; May S.; et al The Relationship between Livestock Ownership and Child Stunting in Three Countries in Eastern Africa Using National Survey Data. PLOS ONE 2015, 10 (9), e0136686 10.1371/journal.pone.0136686 26361393 PMC4567267

